# Expression of the heat shock gene *clpL *of *Streptococcus thermophilus *is induced by both heat and cold shock

**DOI:** 10.1186/1475-2859-5-6

**Published:** 2006-02-15

**Authors:** Mario Varcamonti, Slavica Arsenijevic, Luca Martirani, Daniela Fusco, Gino Naclerio, Maurilio De Felice

**Affiliations:** 1 Dept. of Structural and Functional Biology, University "Federico II", via Mezzocannone 16, 80134 Naples, Italy; 2 Laboratory of Molecular Microbiology and Biotechnology, Department of Molecular Biology, University of Siena, Italy; 3 Department of Experimental Pathology, Section on Microbiology and Virology, University of Bologna, Italy; 4 Faculty of Science, University of Molise, via Mazzini 8, 86170 Isernia, Italy

## Abstract

**Background:**

Heat and cold shock response are normally considered as independent phenomena. A small amount of evidence suggests instead that interactions may exist between them in two *Lactococcus *strains.

**Results:**

We show the occurrence of molecular relationships between the mechanisms of cold and heat adaptations in *Streptococcus thermophilus*, a lactic acid bacterium widely used in dairy fermentation, where it undergoes both types of stress. We observed that cryotolerance is increased when cells are pre-incubated at high temperature. In addition, the production of a protein, identified as ClpL, a member of the heat-shock ATPase family Clp A/B, is induced at both high and low temperature. A knock-out *clpL *mutant is deficient in both heat and cold tolerance. However lack of production of this protein does not abolish the positive effect of heat pre-treatment towards cryotolerance.

**Conclusion:**

Dual induction of ClpL by cold and heat exposure of cells and reduced tolerance to both temperature shocks in a *clpL *mutant indicates that the two stress responses are correlated in *S. thermophilus*. However this protein is not responsible by itself for cryotolerance of cells pre-treated at high temperature, indicating that ClpL is necessary for the two phenomena, but does not account by itself for the relationships between them.

## Background

Although cold and heat shock are normally considered as independent processes, recent evidence points to the occurrence of interlock between them is some organisms. Physiological experiments suggest that cold-shock enhances heat tolerance of *Lactococcus lactis *sp. *lactis *IL1403 [1]. Possible correlations between the heat and cold shock regulons were suggested by i) the observation of an increased level of *Leuconostoc mesenteroides *homologues of the heat shock proteins DnaK and GroEL upon cold-shock [2], ii) the cold induction of a group of small heat shock genes in *Lactobacillus plantarum *[3,4] and iii) a slight protection from freezing of *Lactobacillus johnsonii *[5] and *Lc. lactis *sp. *lactis *[6] cells upon induction of the heat-shock response. Conversely, heat-shock did not improve cryotolerance of *Lc lactis *sp. *cremoris *strain MG1363 [7], suggesting a strain specificity of this phenomenon.

An unidentified cold-inducible 45-kDa protein of *Lc lactis *[8] was proposed to correspond to the heat-inducible ClpX ATPase [9], a member of the large family of closely related ATPases found in both prokaryotic and eukaryotic cells [10], having multiple regulatory functions, included a general chaperone activity and the ability to enable the Clp proteases to recognize their substrates [11,12]. Thus a ClpX function may be to promote the proteolysis of mis-folded proteins after both cold or heat-shock.

In order to gain a deeper insight into the correlation between cold and heat shock, we analyzed the phenomenon in *Streptococcus thermophilus*, a moderate thermophilic LAB widely used in dairy fermentation, where cold and heat stresses are common. Here we show that the synthesis of a 75 kDa protein is induced in both conditions and this induction is essential for stress tolerance.

## Results and discussion

### Influence of heat and cold shock on *S. thermophilus *cryotolerance

**Figure 1:**
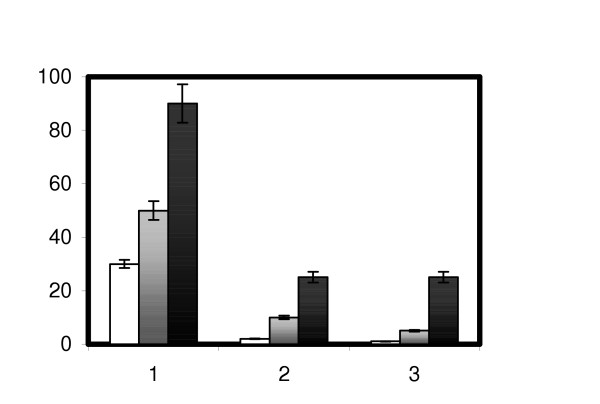
Survival of *S. thermophilus *SFi39 after freezing upon direct freezing from 42°C (white bar), heat-shock pre-adaptation (gray bar) and cold-shock pre-adaptation (black bar). Survival (y-axis) is expressed as the percentage of surviving cells compared to the number of cells prior to freezing (100). Cells survival was tested one, two and three days (x-axis) after freezing. Error bars indicate the coefficient of variation in different experiments.

In order to investigate whether cryotolerance of *S. thermophilus *cells during storage is improved by cold- and/or heat-shock, aliquots of cells grown to middle exponential phase at 42°C were exposed to high and low temperature shocks (30 min at 50°C and 4 h at 20°C, respectively), then fractions of live cells were analyzed after one, two and three days of freeze challenge. Fig. 1 shows that cold shock treatment gives a high protection to freezing, and, in analogy to observations with *Lb. johnsonii *[5] and *Lc. lactis *sp. *lactis *[6], an induction of the heat-shock response gives a slight protection from freezing.

Enhanced cryotolerance was not detected when erythromycin was present at sublethal concentration (2 μg/ml) during heat and cold shock treatments (not shown), which indicated that *de novo *synthesis of proteins is essential for freezing survival. This is not surprising, since, for example, it is known that GroEL and DnaK are induced by heat and cold stress in other LAB [4] and are involved in heat and cold tolerance of *Escherichia coli *[13].

### Heat and cold induction of a 75 kDa protein

**Figure 2:**
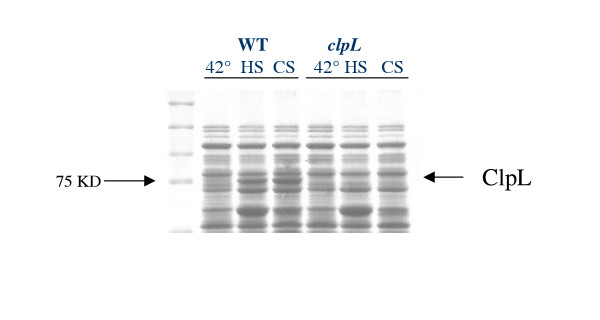
SDS-PAGE of cell extracts of *S. thermophilus *SFi39, wild type and *clpL*Δ, after growth at 42°C (lanes 1 and 4), after heat shock induction (lanes 2 and 5) and cold shock induction (lanes 3 and 6). Equal amounts of protein were loaded on the gel.

**Figure 3:**
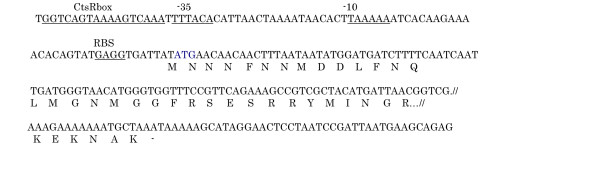
Promoter region and N-terminal domain of ClpL. The CtsR box, promoter sequences and putative RBS are underlined. The first nucleotide transcribed and the N-terminal amino acids sequenced are in bold.

In order to look for proteins induced by heat and cold stresses, we performed a protein extraction followed by SDS PAGE after exposure of *S. thermophilus *cells to the two temperature shocks. In addition to a few bands most likely corresponding to well known proteins accumulated during the early phase of the heat shock response, a band of apparent molecular mass of 75 kDa was induced by both heat and cold (fig. 2). The two bands were eluted from the HS and CS lanes and N-terminal sequenced. The first 13 amino acids (MNNNFNNMDDLFN) were the same for both and were identical to those of the ClpL protein of *S. thermophilus *[14] and to a previously reported 75 kDa heat shock induced protein [15]. Based on the N-terminal 10 amino acids of the identified protein, we synthesized a 30 b oligonucleotide that was used to perform Southern hybridization with the *Kpn*I-digested SFi39 chromosome. A 1500 bp fragment was isolated, cloned and sequenced; one 300 aa ORF starting with the expected 13 amino acids was identified (fig. 3) and resulted 99% identical to the class III heat shock protein ClpL of *S. thermophilus CNRZ1066*, a member of the ClpA/B ATPase family [16].

Most class III heat shock genes are controlled by the class III stress gene repressor CtsR, which binds to a specific direct repeat referred to as CtsR-box [17]. The promoter region of many *clp *genes contains a sequence homologous to the CtsR box (a directly repeated heptanucleotide, A/GGTCAAANANA/G GTCAAA) [17].

In other LAB, CtsR operators were also found upstream of several *clp *genes (*L. sakei *and *S. salivarius*) [17] and *clpP*, *clpE*, *clpL*, *cstR-clpC *genes of *S. pneumoniae *[18] and other Hsp encoding genes, *Lo18 *of *Oenococcus oeni, hsp16 *of *S. thermophilus *[17] and *groESL *of *S. pneumoniae *[18]. A putative CtsR consensus sequence is also present in the promoter region of the Sfi39 *clpL *gene (fig. 3), 44 base pairs upstream of the first transcribed nucleotide (identified by primer extension analysis, data not shown).

### *clpL *disruption reduces tolerance to heat and cold shock

In order to understand the function of ClpL in *S. thermophilus*, we constructed a SFi39 mutant by cloning an internal fragment of the gene in the pG+host9 vector [19]. SFi39 cells were transformed with the recombinant plasmid and forced for homologous recombination of the plasmid into the chromosome. Chromosomal DNA isolated from both wild-type and erythromycin-resistant cells was analyzed by Southern blotting using a specific *clpL *sequence as a probe. The absence of gene product in the mutant was confirmed by showing that the ClpL band present in the wild type was not present in the transformed strain (fig. 2). The mutant obtained was named *clpL*Δ.

**Figure 4:**
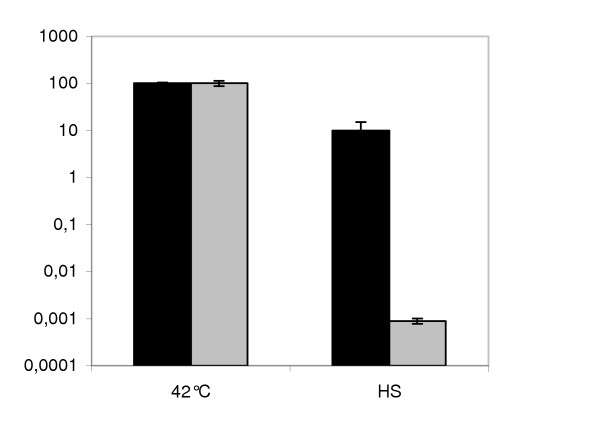
*Streptococcus thermophilus *SFi39 (black bars) and *clpL*Δ (gray bars) survival upon induction of heat shock (HS). Survival is reported as the percentage (y-axis) of colony-forming cells after the treatment compared to that after growth at 42°C (100%). Error bars indicate the coefficient of variation in three different experiments.

Cells from the two strains were grown to middle exponential phase at 42°C. No difference in growth rate was observed in these conditions. The cells were then heat-shocked at 60°C for 1 hour. As shown in fig. 4, *clpL*Δ cells were less tolerant to heat-shock: one wild type cell out of ten survived to the treatment, whereas mutant survival decreased dramatically to 1 cell out of 100,000. This result demonstrates that ClpL, in analogy to well known proteins such as GroEL and DnaK, is involved in the protection of cells against heat.

**Figure 5:**
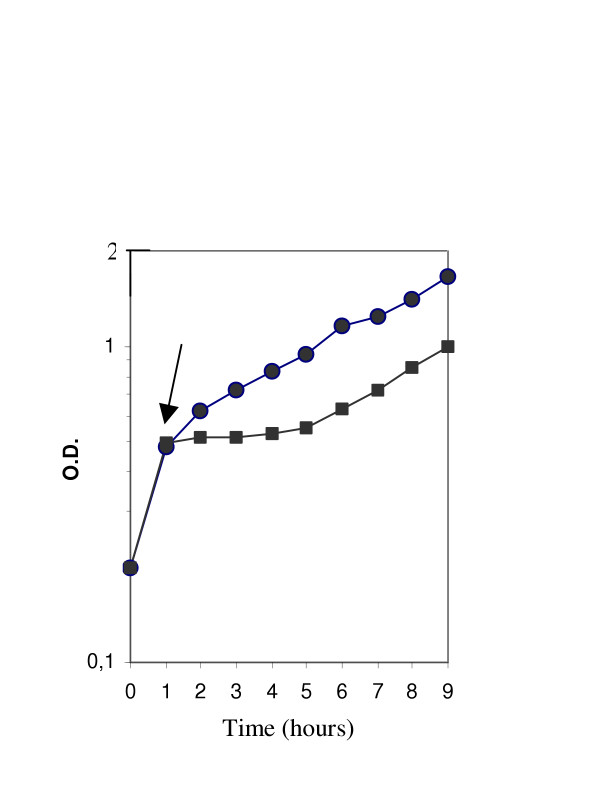
Growth curves of SFi39 (circles) and *clpL*Δ (squares). After the first hour of incubation at 42°C (arrow) cells were incubated at 20°C.

Cold shock tolerance at 20°C of the mutant was tested by measuring its growth, compared to the wild type, after temperature downshift of cells pre-grown exponentially at 42°C. As shown in fig. 5, growth of the wild type continued after temperature downshift at a reduced rate as expected, whereas the *clpL *mutant had to face a long lag phase before restoration of growth at a rate comparable to that of the wild type, which suggests that ClpL is required for normal response to cold shock.

The slight protection from freezing of previously heat-shocked wild type cells shown in fig. 1 was observed also with the ClpL mutant (data not shown), which indicates that the molecular relationship between cold and heat stresses is a complex phenomenon in which the ClpL protein plays only part of the role.

## Conclusion

It is well established that bacteria display adaptive systems to adjust their metabolism to cold and heat shocks. We show that pre-incubation of *S. thermophilus *cells at both high and low temperature enhances their resistance to freezing conditions, which supports the idea that cold and heat shocks, normally considered as unrelated phenomena, have in fact some relationships. At least one shock-induced factor, the ClpL protein, is involved in both phenomena, since we show that i) its synthesis is enhanced in both conditions and ii) no correct response to either stress is observed in a *clpL *knock-out mutant. The finding that this protein is not responsible by itself for the observed phenomenon of cryotolerance of cells pretreated at high temperature indicates that ClpL, although necessary for correct response to both heat and cold stressess, does not account by itself for the moleculr relationships between them, which are most likely based on a more complex regulatory network.

## Methods

### Bacterial strains and growth conditions

*S. thermophilus *SFi39 was cultured at 42°C in M17 broth (Oxoid) containing 1% lactose. Growth was monitored by measuring the optical density at 600 nm (OD600) with a JAS.CO spectrophotometer (model 7800). Erythromycin was added at a concentration of 4 μg/ml. *E. coli *DH5α and EC101 were used as host strains in cloning experiments and were grown in Tryptone Yeast (TY) medium with aeration at 37°C [20]. Ampicillin and erythromycin were used at concentrations of 50 and 100 μg/ml, respectively. To study growth kinetics, 1% inoculated cultures were grown at different temperatures. Growth was monitored by measuring the optical density at 600 nm (OD600).

### Freeze-thaw challenge

To study the freeze-thaw survival capacity, with or without preadaptation, *S. thermophilus *cells were quickly frozen at middle exponential phase (OD600 = 0.5), after heat (30 min at 50°C) and cold shock (4 h at 20°C). Aliquots (1 ml) were spun down (5 min at 6000 rpm), resuspended in 1 ml of fresh LM17 medium, subsequently frozen at -20°C for 24 h, and thawed for 4 min at 30°C in a water bath. The number of CFU was determined just before freezing and after three consecutive freeze-thaw challenges by spread plating decimal dilutions. After 2-day incubations on LM17 plates at 42°C the numbers of CFU were counted. The experiments were performed in duplicate. Coefficient of variation was < 10 %.

### Protein extraction and protein analysis by SDS-PAGE gel electrophoresis

Protein extracts were obtained according to [21] and protein concentration was determined by the "BIO-RAD protein assay" method. Protein analysis was performed using SDS PAGE. Equal amounts of protein (15 μg) were applied on the protein gels and the protein content of the extract was determined using Coomassie Brilliant Blue.

### N-terminal sequencing

500 μg of protein was loaded on SDS-PAGE gel for detection of the N-terminus of specific bands using conditions identical to the analytical gels. The proteins were blotted on a PVDF membrane using a Trans blot unit according to the instruction of the manufacturer (Biorad, Richmond, USA). The PVDF membrane containing separated proteins was stained 1 min with Coomassie Brilliant Blue R-250 (0.1% in water containing 50% methanol), destained for about 10 min in a solution of 40% methanol plus 10% acidic acid in water, and washed in water. Stained protein band was cut from the membrane and subjected to the Edman procedure. BlastP  was used to search for similarity in public protein databases.

### Southern hybridization with degenerated oligonucleotide

Based on the 10 N-terminal amino acids of the isolated protein (MNNNFNNMDD) a 30 b degenerated oligonucleotide was synthesized (5'-ATGAAYAAYAAYTTYAAYAAYATGGAYGAY-3'; Y=T/C) and used in Southern hybridization with chromosomal DNA. Southern hybridization was performed at 37°C overnight, and the membrane was washed 2 min at 37°C in 3XSSC, 0.1% SDS and 2 min at 37°C in 2XSSC, 0.1% SDS, before exposure to X Ray film [17]. The probe was labeled with polynucleotide kinase (Pharmacia, Biotech) ([γ-32P]-ATP.

### DNA manipulations

Chromosomal *S. thermophilus *DNA was isolated as described previously [22]. Cells were transformed by electroporation. *E. coli *cells were transformed by the CaCl2 procedure and plasmid isolation was carried out according to standard procedures [20]. Restriction enzymes, T4 DNA ligase and other DNA-modifying enzymes were purchased from Gibco-BRL Life Technologies, New England Biolabs or Promega, and used as recommended by the manifacturers. Cloning procedures, radiolabelling of DNA fragments, agarose gel electrophoresis and Southern-blot hybridization were performed according to standard procedures [20]. DNA fragments were isolated from agarose gels by using the QIAquick gel extraction Kit (QIAGEN).

### Construction of the *clpL *insertional mutant

To construct an insertional mutant with a disruption in the *S. thermophilus clpL *gene, a 1500 bp PCR fragment (HCIDWF primer: 5'-CTTTTCAATCAATTG ATGGG-3'; HCIDEL482 primer: CATTTGWGAWACWGGRATWCCWGTCAT-3') was amplifed and cloned in the pG+host9 integrational vector [19] digested with *Eco*RI-*Spe*I and the resulting plasmid was designated pGh482. 1 × 108 *S. thermophilus *competent cells were mixed with 500 ng of plasmid pGh482 and electroporated [23]. Transformed cells were grown in anaerobic conditions in the presence of 4 μg erythromycin ml-1 at permissive temperature (30°C), to allow plasmid replication. Integration of pGh482 into the *S. thermophilus *chromosome was forced by growing cells at 30°C up to 0.2–0.3 OD600, then shifting to 42°C and allowing cell growth for two generations (up to 1.2–1.5 OD600). Cells were then diluted and plated on LM17 plates supplemented with 4 μg erythromycin ml-1 and incubated anaerobically at 42°C.

### Stress tolerance

For heat shock treatment, exponentially growing cells (OD600 = 0.5) of Sfi39 and *clpL *mutant were transferred to 60°C for 1 h. Cell survival was measured by plating on LM17 at 42°C before and after incubation at 60°C. For cold-shock treatments 50 ml cultures of Sfi39 and *clpL *mutant were grown in LM17 medium to middle exponential phase (OD600 = 0.5), after which 25 ml of the culture were pelleted (10 min × 4000 g) and re-suspended in the same volume of pre-cooled medium (20°C). Cultures were incubated at 20°C and OD600 values were measured.

## Authors' contributions

MV had a predominant role in the design and implementation of the study and in the preparation of the manuscript, SA performed the identification and cloning of the *clpL *gene and the construction of *S. thermophilus *mutant, LM carried out the protein preparation for Edman sequencing, DF and GN partecipated in the characterization of *clpL *mutant phenotype, MDF helped with discussions and suggestions during the work, preparation of the manuscript and funding. All authors read and approved the final manuscript.
